# Effect of bulk-curing barn insulation on physiological metabolism and energy consumption during flue-cured tobacco curing in cooler-growing regions

**DOI:** 10.3389/fpls.2026.1930629

**Published:** 2026-10-02

**Authors:** Jian’an Wang, Weidong Duan, Zhizhong Zhu, Linlin Zhang, Shunlong Zhai, Guangzhou Cui

**Affiliations:** 1 College of Tobacco Science, Henan Agricultural University, Zhengzhou, China; 2 Tobacco Production Department, China Tobacco Henan Industrial Co., Ltd., Zhengzhou, China; 3 Sales Department, Zhengzhou Gongda High-tech Materials Technology Co., Ltd., Zhengzhou, China; 4 Production Technology Department, Xinyang Branch Company of Henan Provincial Tobacco Company, Xinyang, China

**Keywords:** bulk curing barn, temperature uniformity, cooler-growing regions, energy consumption, insulating materials, pigment degradation

## Abstract

**Introduction:**

Traditional bulk curing barns built with brick and concrete have poor insulation and high energy use during the tobacco curing season—a problem especially pronounced in cooler regions. This study systematically examined how adding new insulation materials to conventional bulk curing barns affects heating performance.

**Methods:**

Two heating systems for bulk curing barns were evaluated: an air-source heat pump (treatments CK1 and T1) and a biomass burner (treatments CK2 and T2).

**Results:**

Barns with added insulation (T1 and T2) significantly improved temperature distribution in the barns’ loading chambers, reducing horizontal and vertical temperature gradients and enhancing overall temperature uniformity. Regarding pigment degradation, adding insulation markedly accelerated chlorophyll loss: total chlorophyll degradation rates for T1 and T2 were 87.30% and 83.63% at 42 °C, respectively—significantly higher than in CK1 and CK2. For biochemical stress markers, malondialdehyde accumulation in T1 and T2 was lower than in the control groups, while polyphenol oxidase activity decreased. Conversely, peroxidase and lipoxygenase activities increased, effectively inhibiting lipid peroxidation. Energy consumption also decreased: T1 and T2 achieved savings of 32.54% and 25.36% in heat consumption, respectively. The payback periods for the retrofit investment in the T1 and T2 curing barns are approximately 2.6 and 1.6 years, respectively.

**Discussio:**

This study demonstrates that adding insulation to bulk curing barns can simultaneously improve the curing environment and increase energy efficiency, particularly in tobacco-growing regions at medium to high altitudes or in barns in poor condition.

## 1 Introduction

Optimizing the curing environment in bulk curing barns (BCBs) for flue-cured tobacco (*Nicotiana tabacum* L.) supports normal physiological metabolism in tobacco leaves and improves leaf quality (Bai et al., 2017). As the primary equipment for flue-cured tobacco production, China has the world’s largest number of BCBs (Wu and Yang, 2021), which collectively consume approximately 2.25 × 10^6^ t of standard coal (tce) annually (Wang et al., 2020). Currently, approximately one million BCBs are operational across China, most of which are brick-and-concrete structures (Wang et al., 2017a). During curing, the temperature inside the BCB gradually increases from 36 °C to 68 °C over approximately 150 h (Wang et al., 2025b). Due to significant heat loss, the thermal insulation of brick-and-concrete buildings is poor. According to statistics, more than 20% of the heat in BCBs is lost to the outside through the walls and roof during tobacco curing, resulting in significant energy losses (Wang et al., 2019).

Altitude plays a significant role in shaping the temperature of the ecological environment (He et al., 2025). As altitude increases, lower ambient temperatures during tobacco harvesting and curing increase heat loss from the external walls of traditional BCBs (Zhai et al., 2025). Simultaneously, the low-temperature environment slows the temperature rise within BCBs, leading to greater temperature differences and longer curing times. This is unfavorable for the yellowing and dehydration of tobacco leaves (Li et al., 2021). These issues, including high energy consumption and low post-curing tobacco quality, undermine the efficiency of local drying facilities (He et al., 2020; Li et al., 2023). Recent studies have shown that establishing optimal curing conditions for tobacco leaves is a key factor in improving curing quality (Wang et al., 2024; Wu et al., 2026a, b).

In modern architectural practice, external wall insulation systems are increasingly important and widespread, shifting from optional features to standard components in new construction (Timuralp et al., 2025; Wang et al., 2025a). Unlike static residential buildings, BCBs operate under dynamic thermal loads and have specific ventilation requirements, making the direct application of building insulation codes challenging (Zhao et al., 2026; Qin et al., 2025). Furthermore, selecting insulation materials for agricultural drying requires balancing thermal conductivity with fire safety, given the proximity to heating equipment that can reach surface temperatures exceeding 500 °C. While some studies have focused on heating equipment upgrades and airflow optimization (Song et al., 2021; Zhang et al., 2022), peer-reviewed research quantifying the comprehensive effects of building envelope retrofits on the internal temperature field, energy consumption, and processing quality under cooler conditions remains scarce.

To address this gap, this study evaluated how thermal insulation retrofits for traditional BCBs affect tobacco leaf physiology during curing—specifically, changes in pigment content and antioxidant enzyme activities. Field experiments compared physiological responses in retrofitted versus conventional barns and analyzed temperature field uniformity, heat-loss characteristics, and specific energy consumption across two heating modes. The findings aim to support low-carbon retrofits of agricultural thermal processing facilities.

## 2 Materials and methods

### 2.1 Experimental materials

Experiments were conducted from June 2023 to September 2025 in tobacco-growing regions (*Nicotiana tabacum* L.) in Lushi County and Pingqiao District, Henan Province, China, and in Gulin County, Sichuan Province, China, at elevations of 923–1220 meters. During the tobacco curing season, the average ambient temperature and relative humidity at the three experimental sites were 20-28 °C and 72-80%, respectively. The tobacco fields were level and had balanced fertility, making them suitable for standardized tobacco cultivation. The tobacco varieties Yunyan 87 and Luxuan 99 were used in this study because their mature leaves are harvested and loaded directly into the BCBs the same day. The experimental BCBs used a conventional local design with an inner forced-downward airflow system. They were constructed of brick-concrete, 30 cm thick, and built in 2013.

The new insulation materials (**Figure 1**) used for T1 and T2 were an inorganic, plasticized, microporous thermal insulation material (IPM) provided by Zhengzhou University. The IPM is a mixture of coal dust, ordinary Portland cement, and a hydrogen peroxide-based industrial foaming agent, a non-combustible, low-toxicity additive commonly used to produce inorganic thermal insulation boards. This material has a volumetric water absorption rate of 4.5%, a dry density of 133 kg/m^3^, a thermal conductivity of 0.046 W/(m·K), a compressive strength of 0.43 MPa, and a tensile strength perpendicular to the panel surface of 124 kPa. Its combustion performance has been certified to meet the A1 non-combustible grade under the Chinese national standard GB 8624-2012, with no flame spread or harmful flue gas emissions when exposed to continuous high temperatures above 800 °C. The selected IPM insulation material in this study has been verified as A1-grade non-combustible, fully meeting the safety requirements for high-temperature operating scenarios.

**Figure 1:**
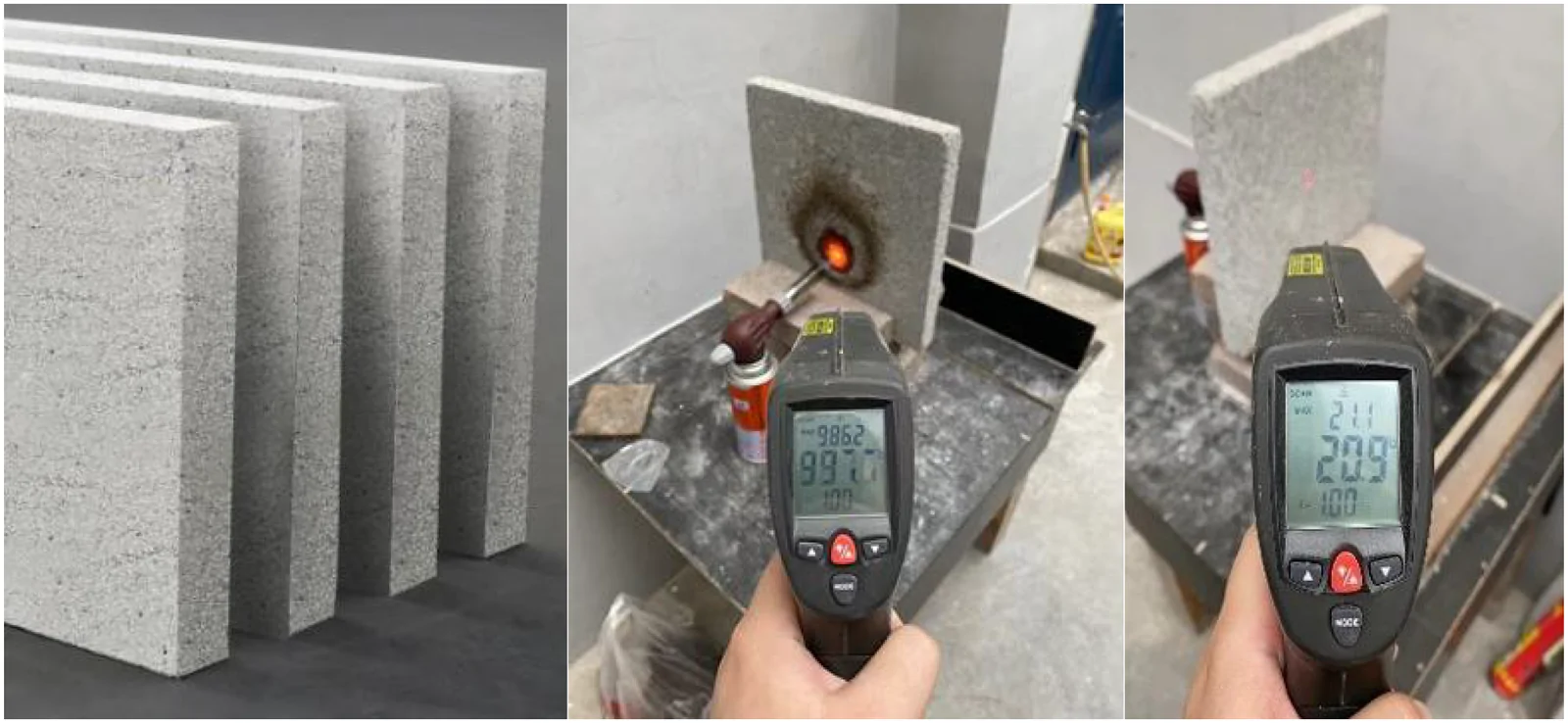
The appearance and thermal insulation test of the inorganic plasticized microporous thermal insulation material. Note: One side was heated to nearly 1,000 °C, while the other side could be touched with the hand.

Twelve independent BCBs with identical brick-concrete structures, service life, and performance parameters were selected for this study, corresponding to the four treatments CK1, T1, CK2, and T2. Each treatment was replicated in 3 completely independent curing batches as biological replicates. All batches were conducted under the same environmental conditions and agronomic background within the same experimental area.

### 2.2 Experimental design

Four treatments (**Table 1**) were designed as follows: CK1 (a BCB with heating from an air-source heat pump), T1 (an insulated, modified version of CK1), CK2 (a BCB with heating from a biomass burner), and T2 (an insulated, modified version of CK2).

**Table 1:** Experimental design.

Treatments	Insulation treatment	Heating equipment of the BCB for tobacco curing	Heating equipment manufacturer
CK1	No insulation renovation	Heating from an air-source heat pump	CSSC Sunrui (Luoyang) Special Equipment Co., Ltd, Luoyang, China
T1	Insulation layer with roofs, walls, and floors	Heating from an air-source heat pump	CSSC Sunrui (Luoyang) Special Equipment Co., Ltd, Luoyang, China
CK2	No insulation renovation	Heating from a biomass burner	Xuchang Tongxing Modern Agricultural Technology Co., Ltd, Xuchang, China
T2	Insulation layer with roofs, walls, and floors	Heating from a biomass burner	Xuchang Tongxing Modern Agricultural Technology Co., Ltd, Xuchang, China

For T1 and T2, a 5 cm-thick IPM insulation layer (provided by Zhengzhou Gongda High-tech Material Technology Co., Ltd.) was installed on the interior surfaces of the roof, walls, and floor. This thickness was chosen to avoid interfering with the loading of subsequent tobacco sticks after the loading chamber was installed. The construction process included bonding the boards with a cement-based adhesive, securing them with expansion anchors, and sealing joints with closed-cell foam to minimize thermal bridging. The floor was excavated to install the insulation layer, then backfilled.

When mature tobacco leaves were harvested and loaded directly into the loading chamber of BCBs on the same day, curing began immediately. The curing process followed local technology standards, including dry-bulb temperature (DBT) and wet-bulb temperature (WBT) for all experimental treatments until completion.

### 2.3 Testing items and methods

#### 2.3.1 Sampling method

For well-developed, middle-positioned tobacco leaves, the 12th leaf counted from the bottom to the top of the tobacco plant was selected as the sampling leaf. The selected leaves must be consistent in size, leaf thickness, and maturity. Every 3 leaves were grouped into one sampling unit, each with a hanging label for identification. A sampling port was set at the middle position of the wall of the loading chamber in BCB, and all sampling units were arranged in sampling order. During the curing process, tobacco leaves were sampled at seven points: the fresh leaf stage (FS) and at curing temperatures of 38 °C, 40 °C, 42 °C, 45 °C, 47 °C, and 54 °C. Immediately after sampling, leaves were frozen in liquid nitrogen for subsequent measurement of pigment content, lipid peroxidation-related enzyme activities, and MDA content. After leaf removal, the resulting gaps were filled with an appropriate amount of burlap to maintain consistent packing density and prevent changes in DBT, WBT, and airflow between leaves that could affect curing performance. All sampling operations were repeated 3 times for each curing batch. The n = 3 indicated in all figure legends in this study represents 3 independent biological replicates obtained from 3 separate curing trials for each treatment, rather than repeated technical measurements of the same sample.

#### 2.3.2 Measurement of the curing barn temperature

The DBT inside the curing barn was recorded using a multi-channel temperature measurement system consisting of a recorder (model: SUP-RN3000, Supmea) and a wired thermoresistor probe (model: SUP-WZP-Y-B, Meilong). The three-tier curing barn contained six data collection zones on each level, as depicted in **Figure 2**. A total of 18 thermoresistor probes were positioned at the intersections of a 4-by-3 grid, formed by dividing the barn’s length into four equal sections and its width into three. To prevent tobacco leaves from contacting or adhering to the 18 thermoresistor probes — which could interfere with DBT data collection — each probe was securely mounted in the center of a dedicated protective cage. After the curing process, the horizontal and vertical DBT differences within each tier of the curing barn were calculated:

**Figure 2:**
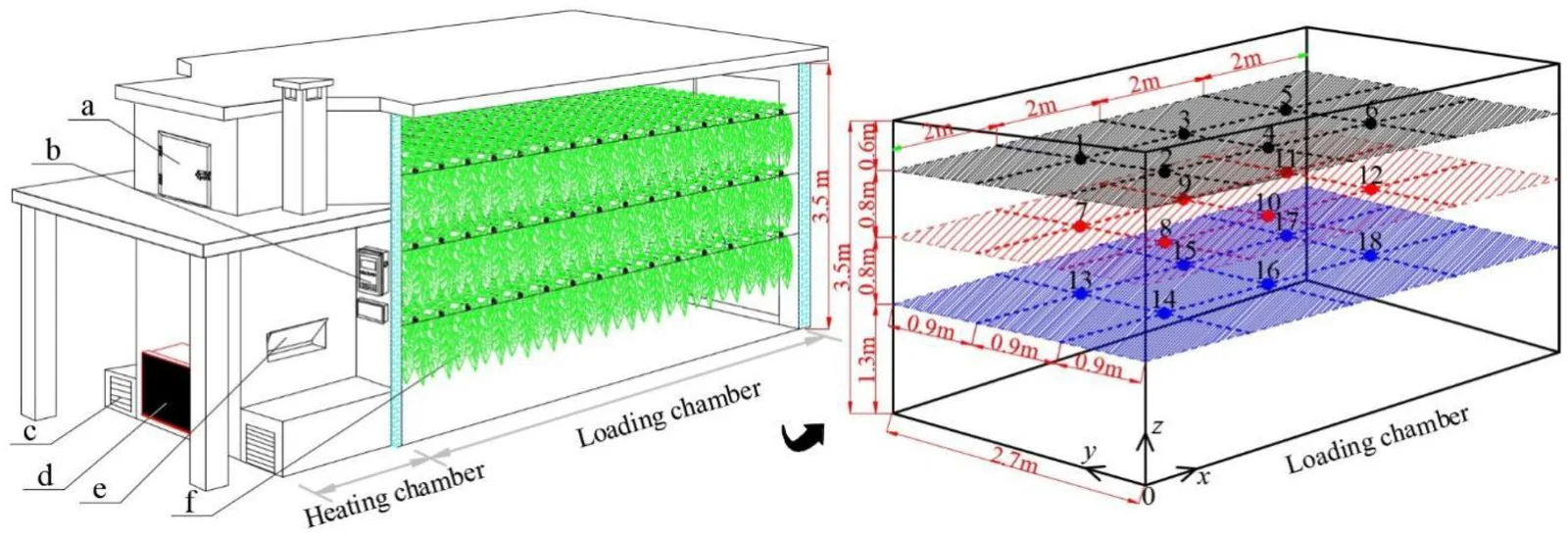
The structure of the experimental BCB and the distribution diagram of the DBT measurement points. Note: **(a)** Repair the circulating fan door, **(b)** BCB controller, **(c)** Moisture discharge outlet, **(d)** Heating equipment (air-source heat pump or biomass burner), **(e)** Door for external fresh air, **(f)** Tobacco leaves. “•” marks the DBT measurement points; “0” denotes the origin of the curing barn’s three-dimensional coordinate system; “x” represents the length of the loading chamber; “y” represents the width; and “z” represents the height.

(1) $$\Delta T_{H}=\frac{\left(T_{i,\max}-T_{i,\min}\right)+\left(T_{ii,\max}-T_{ii,\min}\right)+\left(T_{iii,\max}-T_{iii,\min}\right)}{3}$$

(2) $$\Delta T_{V}=\frac{\left(T_{i-1}-T_{iii-1}\right)+\left(T_{i-2}-T_{iii-2}\right)+\ldots +\left(T_{i-6}-T_{iii-6}\right)}{6}$$

Where: *ΔTH*: horizontal DBT difference, °C; *ΔTV*: vertical DBT difference, °C; *T_i_*_,max_, …, *T_iii_*_,min_, and T*_i-1_*, T*_i-2_*, …, T*_i-6_*: specific DBT values, °C; The subscripts *i*, *ii*, and *iii* represent the lower, middle, and upper tiers of the curing barn, respectively; The subscripts *1*, *2*, …, *6* denote the position numbers of sensors arranged on each tier (**Figure 2**); max and min denote the maximum and minimum DBT values of all sensors on the corresponding tier, respectively.

The horizontal and vertical reduction rates for the DBT difference were calculated using the following formulas:

(3) $$H_{\mathrm{Rr}}=\frac{\left({\mathrm{\Delta T}}_{H,CKx}-{\mathrm{\Delta T}}_{H,Tx}\right)}{{\mathrm{\Delta T}}_{H,CKx}}\times 100\%$$

(4) $$V_{\mathrm{Rr}}=\frac{\left({\mathrm{\Delta T}}_{V,CKx}-{\mathrm{\Delta T}}_{V,Tx}\right)}{{\mathrm{\Delta T}}_{V,CKx}}\times 100\%$$

Where: *H_Rr_*: Horizontal reduction rate of the DBT difference, %; *ΔT_H, CKx_*: Horizontal DBT difference for CK1 or CK2, °C; *ΔT_H, Tx_*: Horizontal DBT difference for T1 or T2, °C; *V_Rr_*: Vertical reduction rate of the DBT difference, %; *ΔT_V, CKx_*: Vertical DBT difference for CK1 or CK2, °C; *ΔT_V, Tx_*: Vertical DBT difference for T1 or T2, °C.

The DBT non-uniformity coefficient was calculated as follows (Bai et al., 2017):

(5) $$U_{x}=\frac{T_{x,\max}-T_{x,\min}}{T_{v,avg}}\times 100\%$$

Where: *U_x_*: DBT non-uniformity coefficient, %; *T_x,_*_max_: Maximum DBT across all measurement points under treatment *x*, °C; *T_x,_*_min_: Minimum DBT across all measurement points under treatment *x*, °C; *T_v,_*_avg_: Average DBT across all measurement points under treatment *x*, °C; *x*: Treatment identifier, corresponding to CK1, CK2, T1, or T2.

The overall DBT uniformity improvement rate was calculated:

(6) $$\eta_{Ux}=\frac{{\bar{U}}_{CKx}-{\bar{U}}_{Tx}}{{\bar{U}}_{CKx}}\times 100\%$$

Where: *η_Ux_*: Overall DBT uniformity improvement rate for BCB, %; ${\bar{U}}_{CKr}$: Average DBT non-uniformity coefficient for the control groups, %; ${\bar{U}}_{Tx}$: Average DBT non-uniformity coefficient for the insulation-modified barns, %.

The energy savings percentage was calculated using the following equation:

(7) $$\eta_{E}=\frac{E_{\mathrm{CK}}-E_{T}}{E_{T}}\times 100\%$$

Where: *η*_E_: Energy-saving percentage for BCB, %; *E*_T_: Heat consumption per unit of dry tobacco weight for the control groups, MJ/kg; *E*_CK_: Heat consumption per unit of dry tobacco weight for the insulation-modified barns, MJ/kg.

Carbon emission reduction performance was calculated:

(8) $$C_{GHG}=B_{GHG}+E_{GHG}$$

Where: C_GHG_ for different fuel types of BCB each year, t CO_2_-eq; BGHG for biomass pellets carbon emission reduction from the renovation of a single biomass-fueled barn or a heat pump barn each year, t CO_2_-eq; E_GHG_ for electricity savings and carbon emission reduction from the renovation of a single biomass-fueled barn or a heat pump barn each year, t CO_2_-eq.

#### 2.3.3 Determination of physiological indicators and pigments

Chlorophyll a, chlorophyll b, total chlorophyll, carotenoids, and abscisic acid (ABA) in tobacco leaves were quantified by high-performance liquid chromatography (HPLC, Agilent 1260 Infinity II, Agilent Technologies, USA). For extraction, 0.5 g of frozen tobacco leaf powder was homogenized in 5 mL of 80% ice-cold acetone, incubated in the dark at 4 °C for 24 h, then centrifuged at 12,000 ×g for 15 min at 4 °C. The supernatant was filtered through a 0.22 μm organic microporous membrane before injection. The HPLC system was equipped with a C18 reversed-phase column (250 mm × 4.6 mm, 5 μm, Agilent ZORBAX SB-C18). The mobile phase consisted of solvent A (methanol) and solvent B (0.1% formic acid aqueous solution) with a gradient elution: 0–10 min, 80% A; 10–20 min, 95% A; 20–25 min, 95% A; 25–30 min, 80% A. The flow rate was set at 1.0 mL/min, the column temperature was maintained at 30 °C, the detection wavelengths were set to 663 nm for chlorophyll, 448 nm for carotenoids, and 265 nm for ABA, and the injection volume was 10 μL. Quantification of each target compound was performed using external standard calibration curves prepared with authentic reference standards (Sigma-Aldrich, USA). The surface color parameters (L, a, b, C, and h) of cured tobacco leaves were measured with a Precision colorimeter (NR110, USA).

Peroxidase (POD) activity was determined using the guaiacol colorimetric method. The crude enzyme extract was prepared by homogenizing 0.5 g of frozen leaf powder in 5 mL of 0.05 mol/L phosphate buffer (pH 6.0, containing 0.1 mmol/L EDTA and 1% (w/v) polyvinylpyrrolidone), followed by centrifugation at 12,000 ×g for 20 min at 4 °C. The reaction mixture contained 2.9 mL of 0.05 mol/L phosphate buffer, 1.0 mL of 2% (v/v) H_2_O_2_, 1.0 mL of 0.05 mol/L guaiacol, and 0.1 mL of the crude enzyme extract. Absorbance at 470 nm was recorded at 30 s intervals for 2 min using a UV spectrophotometer (UV-1800, Shimadzu, Japan). One unit of POD activity was defined as the amount of enzyme that produced a 0.01 increase in absorbance at 470 nm per minute per gram of fresh sample.

Polyphenol oxidase (PPO) activity was measured by the catechol oxidation method. The enzyme extract was prepared in the same phosphate buffer used for the POD assay. The reaction mixture contained 1.5 mL of 0.1 mol/L catechol solution (prepared in pH 6.0 phosphate buffer), 1.5 mL of 0.05 mol/L phosphate buffer, and 0.5 mL of crude enzyme extract. The mixture was incubated at 37 °C for 5 min, and the reaction was terminated by adding 2 mL of 20% trichloroacetic acid. After centrifugation, the absorbance of the supernatant was measured at 420 nm using a UV spectrophotometer (UV-1800, Shimadzu, Japan). One unit of PPO activity was defined as the amount of enzyme that produced a 0.001 increase in absorbance at 420 nm per minute per gram of fresh weight.

Lipoxygenase (LOX) activity was assessed using the linoleic acid oxidation method. Enzyme extraction followed the protocol described in the literature (Li et al., 2018). The reaction mixture contained 2.7 mL of 0.05 mol/L sodium acetate buffer (pH 5.5), 0.2 mL of 10 mmol/L linoleic acid substrate solution, and 0.1 mL of crude enzyme extract. Absorbance at 234 nm was monitored continuously for 3 min using a UV spectrophotometer (UV-1800, Shimadzu, Japan). One unit of LOX activity was defined as the amount of enzyme that produced a 0.01 increase in absorbance at 234 nm per minute per gram of fresh tobacco leaf sample.

Malondialdehyde (MDA) content was determined by the thiobarbituric acid (TBA) method. Briefly, 0.5 g of a frozen leaf sample was homogenized in 5 mL of 10% (w/v) trichloroacetic acid (TCA) and centrifuged at 4,000 ×g for 10 min. A 2 mL aliquot of the supernatant was mixed with 2 mL of 0.67% TBA solution (prepared in 10% TCA), then boiled in a water bath for 30 min and immediately cooled to room temperature to terminate the reaction. After a second centrifugation, the absorbance of the supernatant was measured at 450 nm, 532 nm, and 600 nm using a UV spectrophotometer (UV-1800, Shimadzu, Japan). The MDA concentration was calculated using the correction formula: MDA concentration (μmol/L) = 6.45 × (A_532_ - A_600_) - 0.56 × A_450_, and the final result was expressed as μmol per gram of fresh leaf weight.

The analysis was conducted by Henan China Tobacco Industry Co., Ltd., and the contents of chlorogenic acid, cryptochlorogenic acid, neochlorogenic acid, scopolamine, and rutin were measured in accordance with the YC/T202–2006 standards.

#### 2.3.4 Determination of the appearance quality of cured tobacco leaves

The appearance quality of cured tobacco leaves was evaluated in accordance with the national standard GB 2635-92, using a 10-point scale to assess color, maturity, leaf structure, oil content, identity, and hue. The total score was calculated using the following weights: 0.20 for color, 0.30 for maturity, 0.16 for leaf structure, 0.12 for oil content, 0.10 for identity, and 0.12 for hue.

#### 2.3.5 Determination of the physical properties of the cured tobacco leaves

The length, width, thickness, single-leaf weight, tensile strength, and equilibrium moisture content of the cured tobacco leaves were measured, and the total score was calculated using the fuzzy evaluation method (Jiang et al., 2024). Each property was measured three times.

#### 2.3.6 Curing barn energy consumption statistics

The tobacco loading amount, biomass, electricity consumption, and curing time for each treatment were recorded and analyzed.

#### 2.3.7 Statistical analysis

All experimental data were organized and initially preprocessed in Microsoft Excel 2021, and statistical analyses were conducted in SPSS 26.0 (IBM Corp., Armonk, NY, USA). Normality of all datasets was assessed with the Shapiro-Wilk test, and homogeneity of variance was evaluated with Levene’s test.

One-way ANOVA was used to compare indicators across treatment groups—CK1, T1, CK2, and T2. When data were normally distributed and had equal variances, Duncan’s multiple range test was used for *post hoc* comparisons to identify significant differences among treatments. If the assumption of homogeneity of variances was violated, Tamhane’s T2 test was used for multiple comparisons.

All significance thresholds in this study were set at *P* < 0.05. Differences with *P* ≤ 0.05 were considered statistically significant, and those with *P* ≤ 0.01 were considered highly significant. Results are presented as mean ± standard error, based on n = 3, representing three independent biological replicates from separate curing trials per treatment, not technical replicates. Pearson correlation coefficients were used to assess relationships among indicators, and the correlation heatmap was generated in OriginPro 2025.

## 3 Results

### 3.1 Changes in tobacco leaf pigments under different treatments

#### 3.1.1 Changes in chlorophyll content under different treatments

As shown in **Figure 3a**, chlorophyll a degradation accelerated markedly at 40 °C. Chlorophyll a content in T1 and T2 was 13.99% and 15.84% lower than in CK1 and CK2, respectively, relative to FS, and the differences were significant (*P* < 0.05). **Figure 3b** shows that between 38 °C and 45 °C, chlorophyll b degradation increased by 10.73 and 6.58 percentage points in T1 and T2, respectively, relative to CK1 and CK2. T1 exhibited the highest degradation rate, followed by T2, with significant differences among treatments (*P* < 0.05).

**Figure 3:**
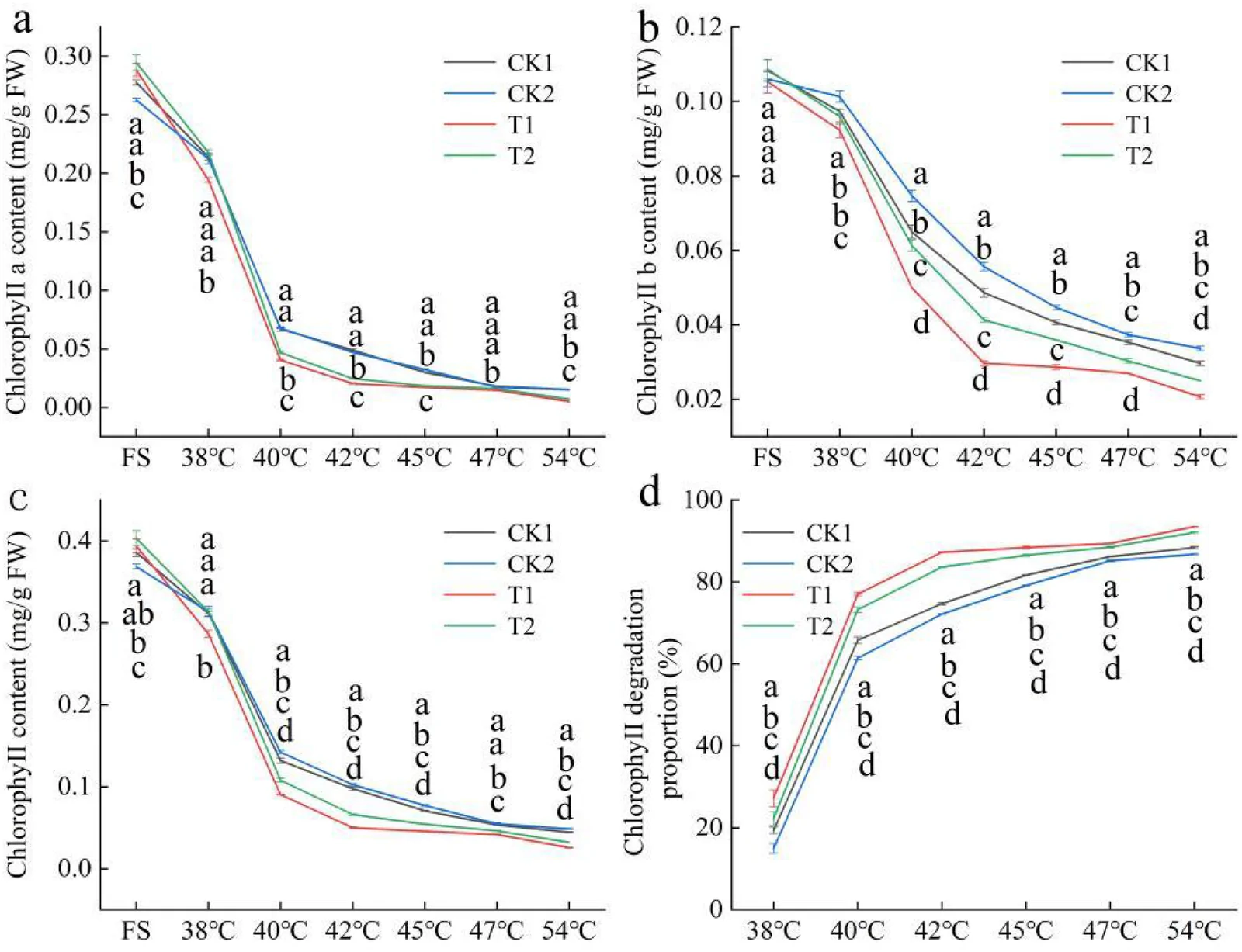
Changes in chlorophyll content across the processing stages of tobacco leaf curing. Note: **(a)** Chlorophyll a content; **(b)** Chlorophyll b degradation rate; **(c)** Total chlorophyll content; **(d)** Total chlorophyll degradation proportion. Different letters indicate significant differences (*P* < 0.05) among treatments within the same curing stage. Data are presented as mean ± standard error, with n = 3 representing 3 independent biological replicates from separate curing trials for each treatment, unless otherwise stated.

As shown in **Figure 3c**, total chlorophyll across all treatments decreased continuously with increasing DBT, consistent with trends in chlorophyll a and b. Degradation occurred primarily between 38 and 45 °C and slowed significantly above 47 °C. Before 42 °C, degradation rates in T1 and T2 were significantly higher than in CK1 and CK2 (*P* < 0.05), though this difference narrowed as DBT increased. At 42 °C, the total chlorophyll degradation rates for T1 and T2 were 87.30% and 83.63%, respectively (**Figure 3d**). This effectively reduces the risk of green-vein or green-stain defects, with significant differences (*P* < 0.05) (Chu et al., 2010).

#### 3.1.2 Changes in carotenoid content under different treatments

As shown in **Figure 4a**, carotenoid content across all treatments decreased continuously with increasing DBT, following a “slow–fast–slow” pattern: mild degradation at 38–40 °C, accelerated degradation at 42–47 °C, and a slowdown above 47 °C. Throughout the process, carotenoid content in T1 and T2 was consistently and significantly lower than in controls at the same DBT (*P* < 0.05), indicating higher degradation rates in insulated-modified barns. **Figure 4b** shows that degradation rates increased continuously with DBT. At 42 °C, rates in T1 and T2 were significantly higher than in controls, and CK1 had the lowest rate (*P* < 0.05). Overall, degradation rates ranked T1 > T2 > CK2 > CK1, consistent with the content differences shown in **Figure 4b** and with established enzymatic degradation pathways.

**Figure 4:**
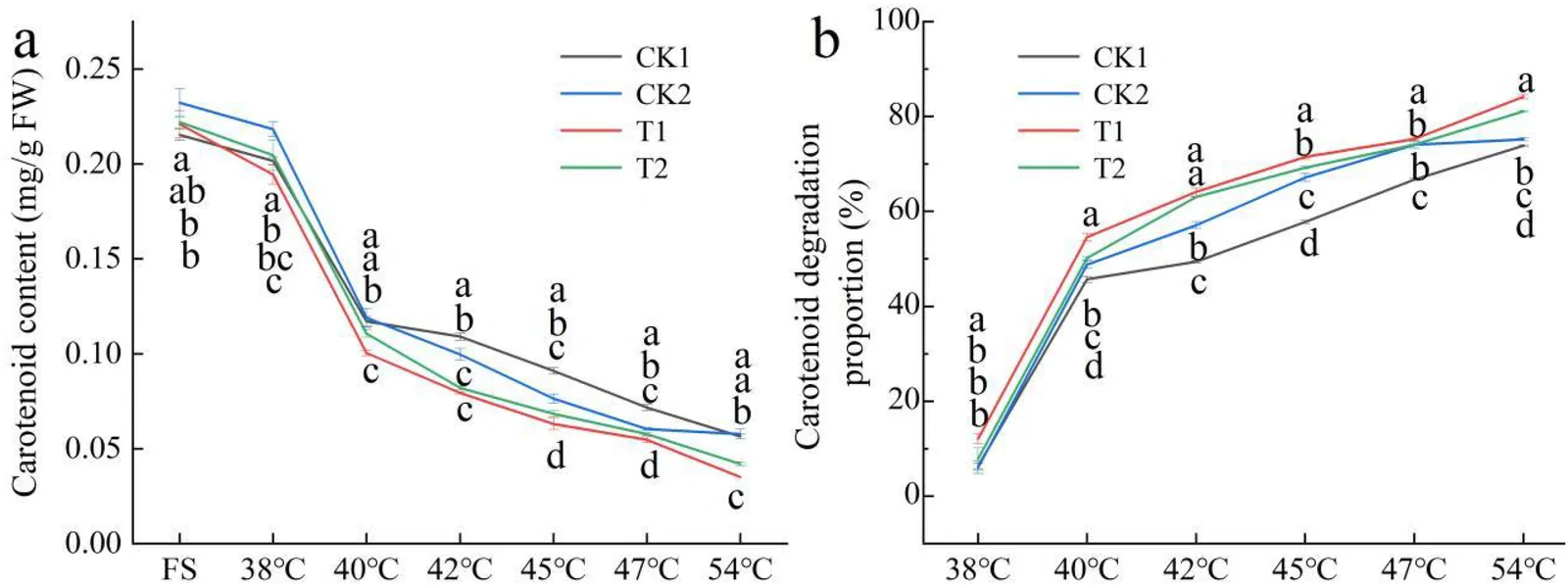
Changes in carotenoid content during curing. **(a)** Carotenoid content; **(b)** Carotenoid degradation proportion. Different letters indicate significant differences (*P* < 0.05) among treatments within the same curing stage. Data are presented as mean ± standard error, with n = 3 representing 3 independent biological replicates from separate curing trials for each treatment, unless otherwise stated.

#### 3.1.3 Comparison of the ratio of carotenoid to chlorophyll under different treatments

The carotenoid-to-chlorophyll ratio in tobacco leaves in T1 and T2 followed an “increase–decrease–increase” pattern. In CK1, the carotenoid-to-chlorophyll ratio initially increased and then decreased with curing temperature, whereas in CK2, it increased as curing temperature rose. Overall, the carotenoid-to-chlorophyll ratio across all treatments showed an upward trend (**Figure 5**). At 42 °C, the carotenoid-to-chlorophyll ratio in T1 was significantly higher than in the other treatments and was generally higher than in CK1 throughout the curing process. The carotenoid-to-chlorophyll ratio in T2 was generally higher than in CK2 throughout the curing process.

**Figure 5:**
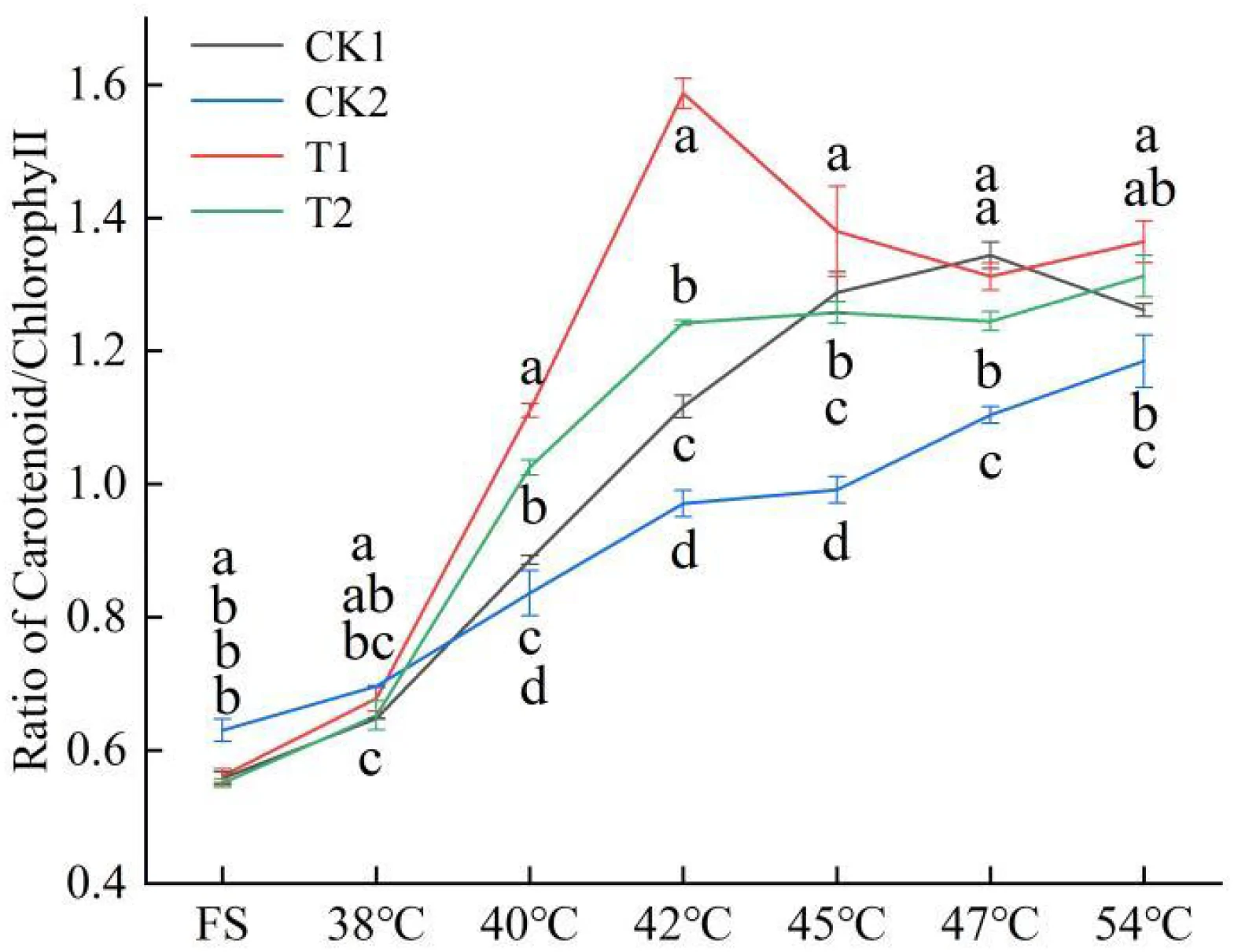
Changes in the ratio of carotenoid to chlorophyll during curing. Different letters indicate significant differences (*P* < 0.05) among treatments within the same curing stage. Data are presented as mean ± standard error, with n = 3 representing 3 independent biological replicates from 3 curing barns.

#### 3.1.4 Comparison of color parameters of tobacco leaves after curing under different treatments

The color parameters L (lightness), a (red-green component), b (yellow-blue component), C (chroma), and h° (hue angle) reflect differences in brightness, redness/greenness, yellowness/blueness, color saturation, and perceived hue of tobacco leaves across treatments, with results summarized in **Table 2**. Regarding lightness (L), T1 and T2 were similar and lower than CK2, indicating that leaves from the insulation-modified barns showed more intense coloration. For the red-green axis (a), T1 and T2 were higher than their corresponding controls, suggesting a greater shift toward red. In terms of yellowness (b), T2 had the highest value, followed by T1 and CK2, with CK1 the lowest, implying that leaves from T2 displayed the strongest yellow dominance. Regarding color saturation (C), T2 had the highest chroma, indicating the most vivid color, whereas CK1 had the lowest, indicating relatively pale coloration. No significant differences in hue angle (h°) were detected among treatments, and all tended toward yellowish hues. As illustrated in **Figure 6**, leaves from T1 and T2 exhibited clearer red-yellow attributes and richer overall color, whereas those from CK1 and CK2 appeared comparatively lighter.

**Table 2:** Comparison of color parameters under different treatments.

Treatments	L	a	b	C	h
CK1	59.75ab	9.85a	51.41b	52.35b	79.15a
T1	56.76b	11.52a	56.24a	57.42a	78.40a
CK2	62.07a	9.72a	55.97a	56.81a	80.14a
T2	56.30b	11.60a	58.19a	59.33a	78.73a

Different letters in the same column indicate significant differences at *P<* 0.05.

**Figure 6:**
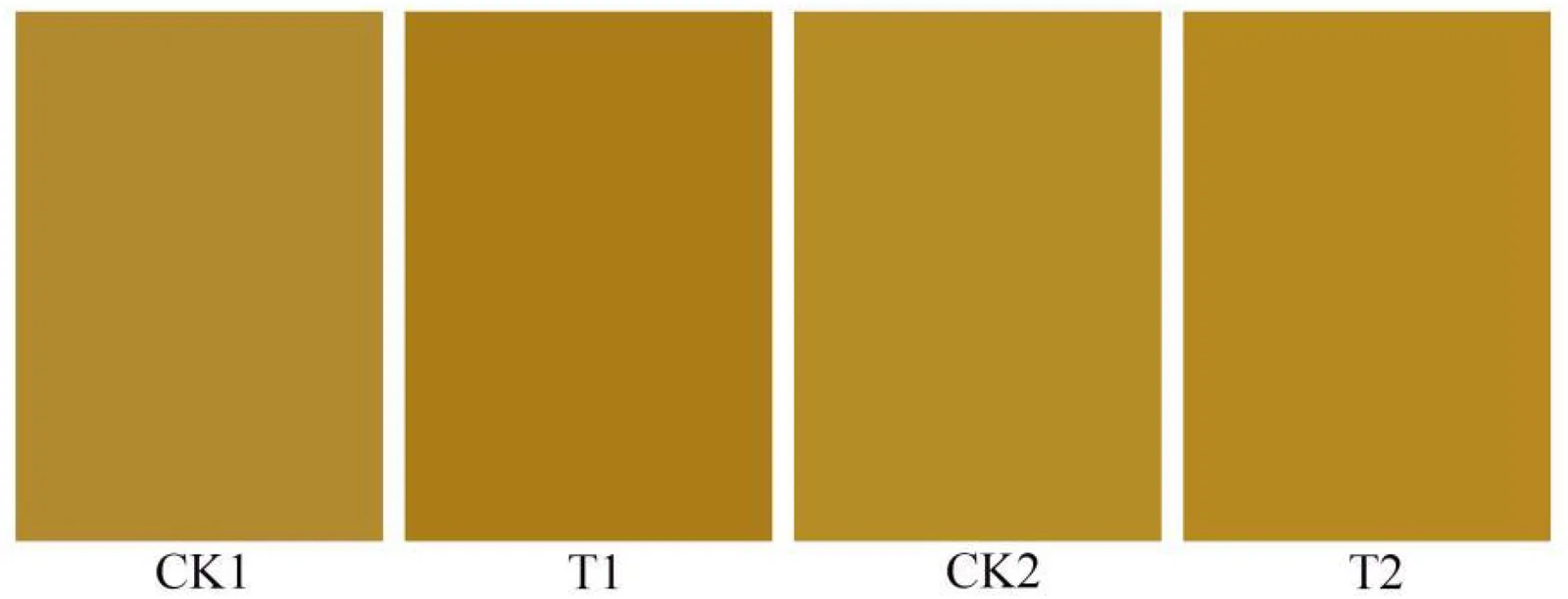
Comparison of standard color cards for cured tobacco leaves across treatments.

### 3.2 Changes in ABA content in tobacco leaves under different treatments

As shown in **Figure 7**, ABA content in all treatments initially increased and then decreased. During early yellowing (FS to 40 °C), ABA accumulation rates in T1 and T2 were significantly higher than in CK1 and CK2. All treatments peaked at 40 °C, with values of 5.65 ± 0.036 μg/g FW (T1), 5.43 ± 0.081 μg/g FW (T2), 3.32 ± 0.095 μg/g FW (CK1), and 4.07 ± 0.180 μg/g FW (CK2). At this point, T1 and T2 were significantly higher than CK1 and CK2 (*P* < 0.05). As DBT rose further, ABA content declined. Between 40 °C and 47 °C, T1 and T2 showed faster degradation rates. At 54 °C, only the difference between CK1 and T1 remained significant (*P* < 0.05). The synchronized ABA-pigment degradation pattern aligns with previous findings on heat-induced metabolic regulation during tobacco curing (Li et al., 2023).

**Figure 7:**
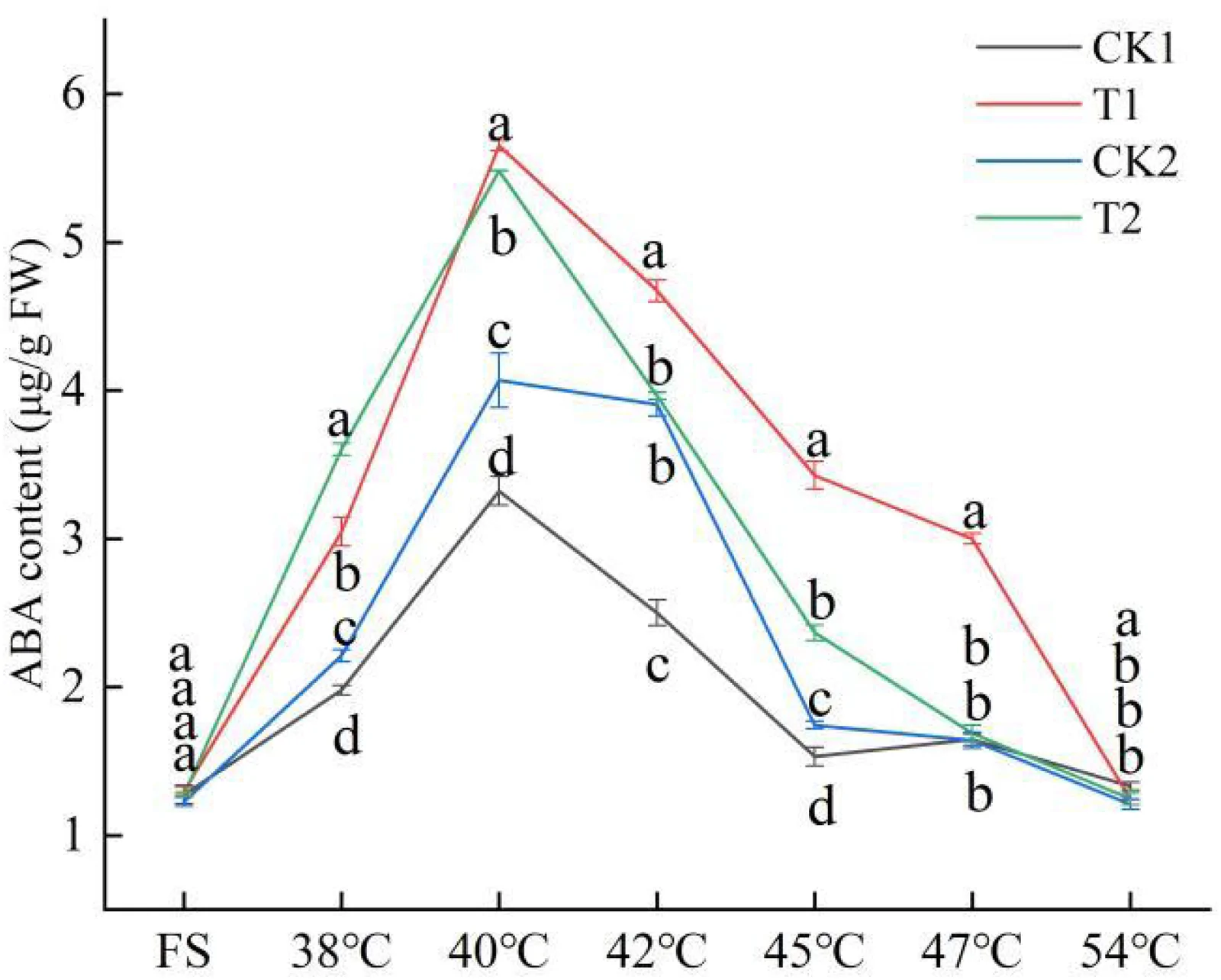
Changes in ABA content during the curing process of tobacco leaves. Different letters indicate significant differences (*P* < 0.05) among treatments within the same curing stage. Data are presented as mean ± standard error, with n = 3 representing 3 independent biological replicates from separate curing trials for each treatment, unless otherwise stated.

### 3.3 Changes in MDA content and enzyme activity of tobacco leaves under different treatments

As shown in **Figure 8a**, MDA content in control groups was positively correlated with DBT, showing an overall upward trend from FS to 54 °C, with the effect particularly pronounced above 47 °C. At any given DBT, MDA content in T1 and T2 was significantly lower than in CK1 and CK2, respectively (*P* < 0.05). Specifically, at 42 °C, MDA accumulation in T1 and T2 was 15.3–19.7% lower than in the corresponding controls. **Figure 8b** shows that PPO activity followed a fluctuating “decrease–then–increase” pattern as DBT increased. At 42–45 °C, PPO activities in CK1 and CK2 were significantly higher than in T1 and T2. Above 47 °C, PPO activity generally declined across all treatments. **Figure 8c** illustrates that POD activity followed a fluctuating “rise-fall” pattern. At 38 °C, POD activities in CK1 and CK2 were significantly higher than in T1 and T2. Conversely, at 40–45 °C, POD activity in T1 and T2 was significantly higher. At 45 °C, a critical phase, higher POD activity reduces the risk of browning; a significant difference was observed between controls and insulation-modified barns (*P* < 0.05). As shown in **Figure 8d**, LOX activity generally followed an “increase–decrease” trend. T1 LOX activity was consistently higher than CK1 across 42–54 °C, particularly at 45 °C. In other phases, T2 and CK2 showed no significant difference. During high-temperature stages, higher LOX activity in T1 and T2 enhances cellular systemic resistance (Hasanuzzaman et al., 2013; Nath et al., 2016).

**Figure 8:**
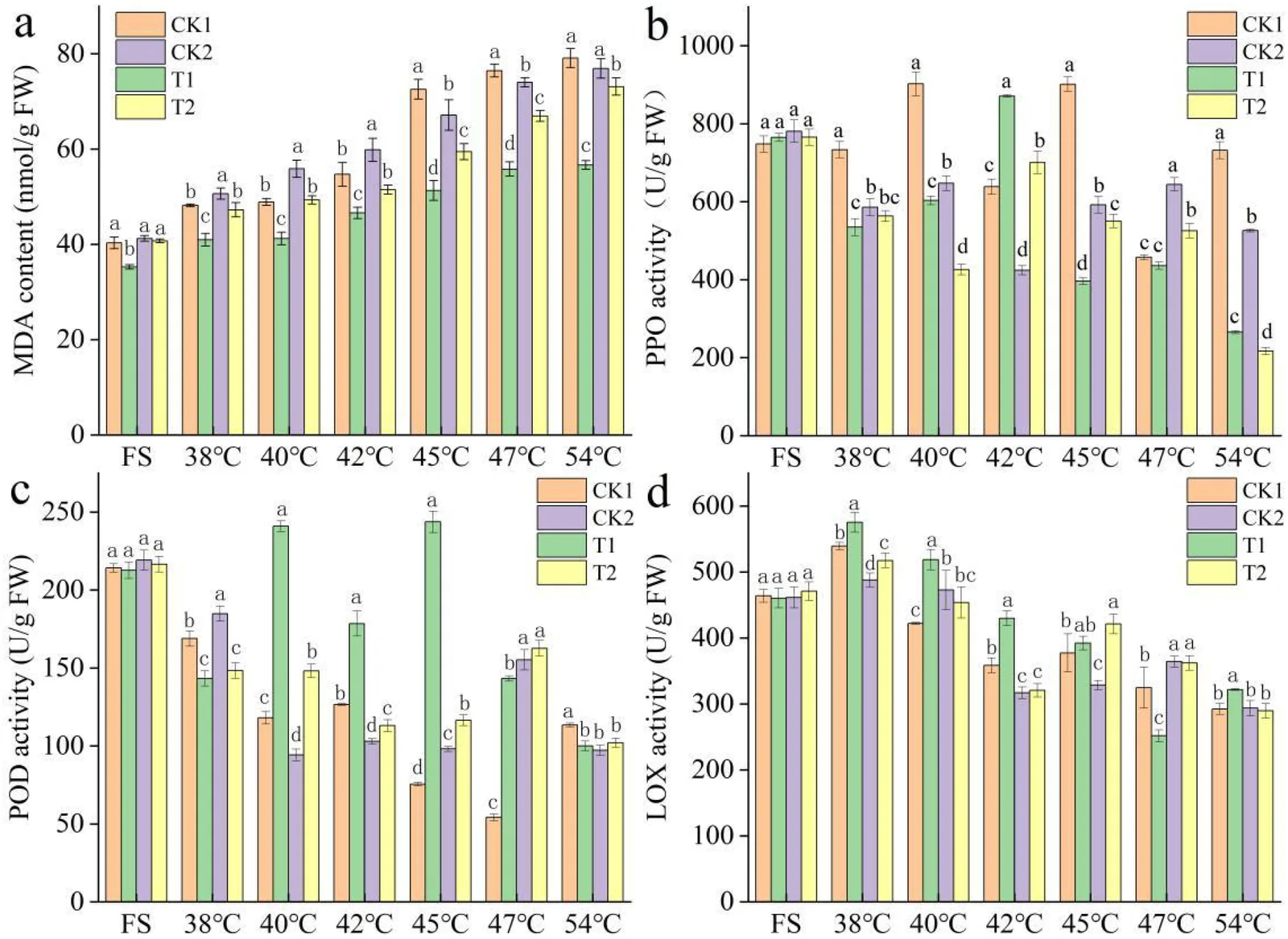
Changes in MDA content and related enzyme activities during tobacco leaf curing. **(a)** MDA content; **(b)** PPO activity; **(c)** POD activity; **(d)** LOX activity. Different letters indicate significant differences (*P* < 0.05) among treatments within the same curing stage. Data are presented as mean ± standard error, with n = 3 representing 3 independent biological replicates from separate curing trials for each treatment, unless otherwise stated.

### 3.4 Comparison of polyphenol content in tobacco leaves after curing under different treatments

As shown in **Table 3**, the contents of neochlorogenic acid, cryptochlorogenic acid, and rutin in T1 and T2 were significantly higher than in the control treatments. The chlorogenic acid content in T2 was significantly higher than in CK2, whereas the chlorogenic acid content in T1 and CK1 was similar. The scopoletin content was comparable across treatments. The polyphenol content followed the order: T2 > CK2 > T1 > CK1. Under the same conditions, the polyphenol content in tobacco leaves from biomass-fueled barns was notably higher than in those from air-source heat pump-heated barns.

**Table 3:** Comparison of polyphenol content in different treatments.

Treatments	Neochlorogenic acid (mg/g)	Chlorogenic acid (mg/g)	Cryptochlorogenic acid (mg/g)	Scopoletin (mg/g)	Rutin (mg/g)	Total (mg/g)
CK1	1.23c	13.23b	2.02c	0.08a	7.15d	23.71c
T1	1.94a	12.70bc	2.75b	0.10a	7.96c	25.45b
CK2	1.60b	11.73c	1.87c	0.09a	10.25b	25.54b
T2	2.00a	16.80a	3.10a	0.10a	13.31a	35.31a

Different letters in the same column indicate significant differences at *P<* 0.05.

### 3.5 Comparison of external quality of tobacco leaves under different treatments

As shown in **Table 4**, the overall appearance quality scores of T1 and T2 were significantly higher than those of CK1 and CK2, primarily reflected in higher scores for leaf structure, integrity, and oil content. Differences in appearance quality between T1 and T2 were mainly observed in color, leaf structure, oil content, and chromaticity. Compared with CK1 and CK2, the leaves in T1 and T2 were looser in structure, richer in oil content, and more intensely colored.

**Table 4:** Comparison of the external quality of tobacco leaves across treatments (score on a 10-point scale).

Treatments	Color	Maturity	Leaf structure	Body	Oil	Chroma	Score
CK1	8.62c	8.71b	7.90d	8.57b	6.76c	8.53c	8.33b
T1	9.07b	9.02a	8.85a	9.41a	7.81a	9.24a	8.96a
CK2	8.22d	8.59b	7.40c	8.33c	6.55d	8.63c	8.10c
T2	9.31a	8.95a	8.56b	9.43a	7.20b	9.01b	8.85a

Different letters in the same column indicate significant differences at *P<* 0.05.

### 3.6 Comparison of physical properties of tobacco leaves under different treatments

As shown in **Table 5**, the total scores for the physical properties of T1 and T2 were significantly higher than those of CK1 and CK2 across heating groups, notable differences in single-leaf weight and equilibrium moisture content. T2 had the largest leaf area, followed by T1, while CK1 had the smallest. Leaf thickness did not differ significantly among treatments. The tensile strength scores of T1 and CK1 did not differ significantly, whereas the tensile strength score of T2 was significantly higher than that of CK2.

**Table 5:** Comparison of physical properties of tobacco leaves under different treatments.

Treatments	Length (cm)	Width (cm)	Weight per leaf (g)	Thickness of leaf (μm)	Equilibrium moisture content (%)	Tensile strength (N)	Score
CK1	61.00b	26.33b	17.94b	86.67a	11.49b	1.55ab	85.63c
T1	65.33a	30.67ab	22.87a	103.00a	13.25a	1.68a	91.18ab
CK2	63.67ab	28.33ab	18.96b	90.67a	10.78b	1.32b	86.89bc
T2	66.67a	32.33a	24.07a	101.00a	12.76a	1.68a	92.91a

Different letters in the same column indicate significant differences at *P* < 0.05.

### 3.7 Correlation analysis of pigment degradation and membrane lipid peroxidation-related enzymes and products

Correlation analysis was conducted among pigment degradation, membrane lipid peroxidation-related enzymes, and product accumulation. All 48 observations in this correlation analysis are independent: they are sourced from 3 biological replicates across 7 sampling stages in the 4 treatments (CK1, T1, CK2, T2), and all observations are derived from separate curing trials rather than repeated technical measurements of the same sample, which fully satisfies the independence assumption of the Pearson correlation analysis method.

As shown in **Figure 9**, the correlation coefficients between ABA content and the degradation of chlorophyll a, chlorophyll b, total chlorophyll, and carotenoids were 0.87, 0.83, 0.87, and 0.85, respectively, all of which were highly significant. Pigment degradation and MDA accumulation showed a significant negative correlation (*P* ≤ 0.05). The activities of POD and LOX enzymes showed highly significant negative correlations with MDA accumulation, with correlation coefficients of -0.70 and -0.73, respectively. The correlation coefficients among LOX enzyme activity, ABA accumulation, and pigment degradation were all > 0.8 and statistically significant at *P* ≤ 0.01. The correlation coefficient between LOX and POD enzyme activities was 0.52, indicating a weak but significant positive relationship at the *P* ≤ 0.05 level.

**Figure 9:**
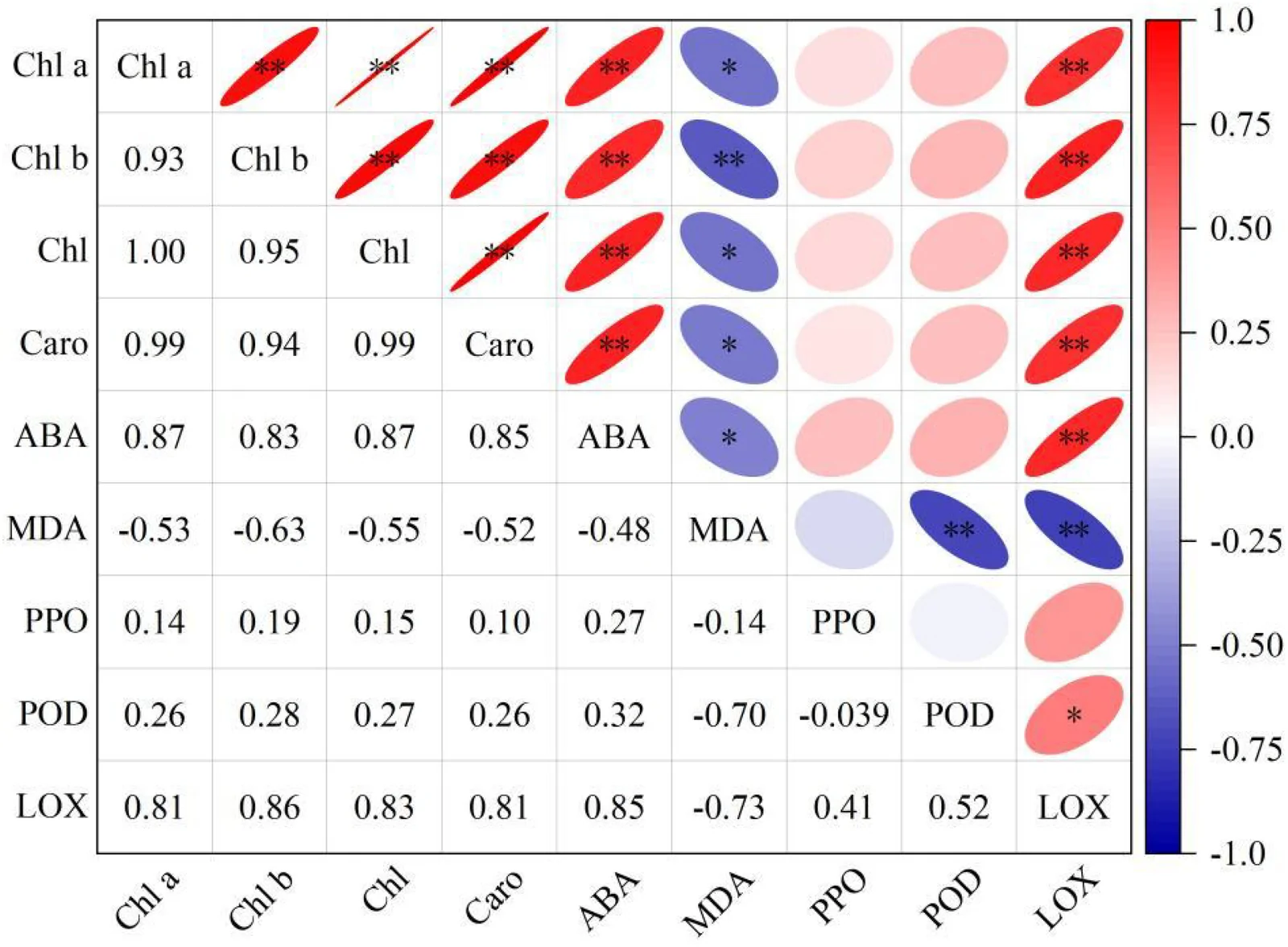
Correlation analysis of pigment degradation and membrane lipid peroxidation-related enzymes and products. Note: This heatmap displays only statistically significant Pearson correlation coefficients between paired indicators; it does not claim or imply any direct causal relationship between any pair of indicators. * denotes significant correlation (*P* ≤ 0.05), ** denotes highly significant correlation (*P* ≤ 0.01). Red indicates a positive correlation between indicators, while blue indicates a negative correlation.

### 3.8 Comparison of curing performance in the curing barn under different treatments

#### 3.8.1 Analysis of temperature field under different treatments

The temperature in the curing barn decreases gradually from the upper tier to the lower tier, with higher temperatures near the barn perimeter. For the same curing duration, the temperature inside the curing barn with T1 is higher than that with CK1, and the temperature in T2 is higher than in CK2. After insulation treatment, the curing barn experiences a faster DBT rise than the control barn. As shown in **Figure 10**, the horizontal and vertical temperature differences in T1 and T2 are smaller, whereas those in CK1 and CK2 are larger. The temperature distribution within the T1 and T2 barns is more uniform, whereas the temperature distribution in the CK1 and CK2 barns is uneven.

**Figure 10:**
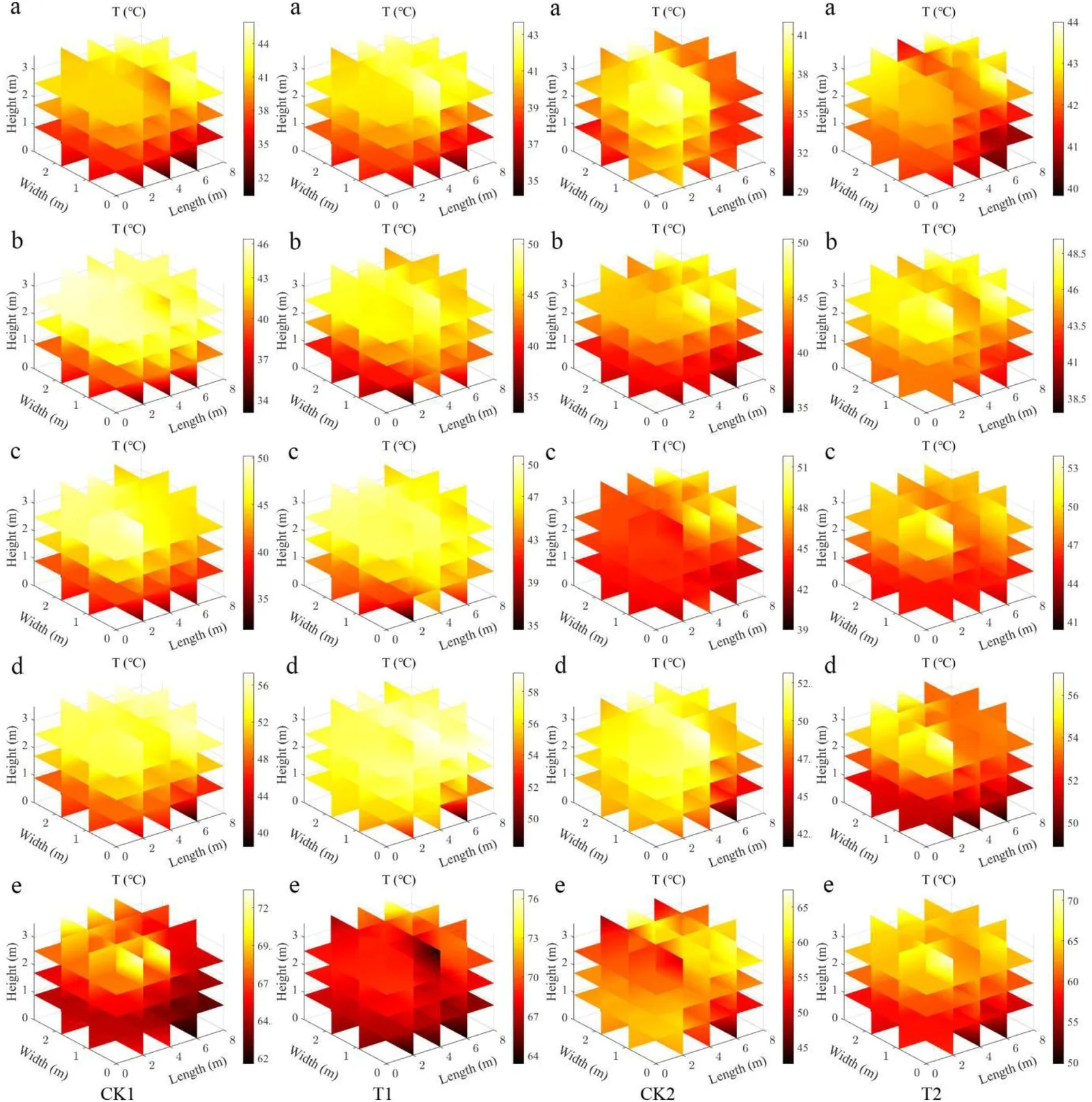
Comparison of temperature fields under different treatments. Note: **(a–e)** represent the curing barn temperatures at 9, 54, 81, 150, and 200 h of curing, respectively.

#### 3.8.2 Comparison of temperature differences in the curing barn under different treatments

As shown in **Figure 11**, the horizontal DBT difference in the upper tiers of T1 and T2, calculated using Equations 1 and 2 during curing, remains stable and relatively low, with the average temperature difference held within 2.06 °C. The average vertical DBT for CK1 and CK2 are 2.92 °C and 3.72 °C, respectively, with CK2 reaching a maximum of 6.50 °C, significantly higher than in the other treatments. Quantitative analysis (Equations 3 to 4) confirmed that the horizontal and vertical DBT differences in the insulated barns were significantly lower than those in the controls. T1 vs CK1: Horizontal gradient decreased by 31.2%, and vertical gradient by 27.6%. T2 vs CK2: Horizontal gradient decreased by 32.5%, and vertical gradient by 28.4%. The overall DBT uniformity improvement rates were 22.4% (T1) and 23.1% (T2), calculated using Equations 5 and 6.

**Figure 11:**
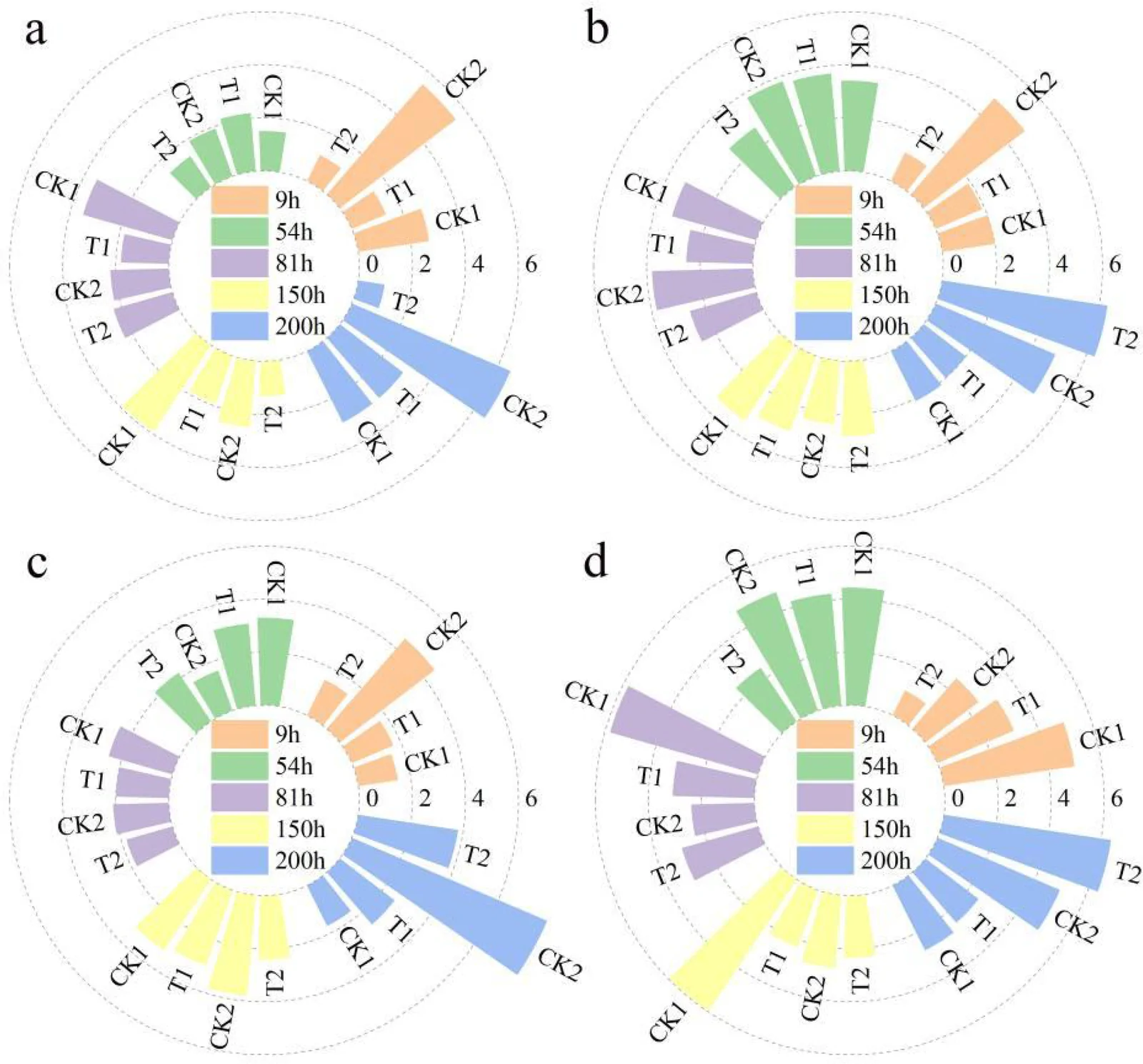
Comparison of temperature differences across treatments. Note: **(a–d)** denote the horizontal and vertical temperature differences in the upper, middle, and lower tiers of the curing barn, with units in °C.

#### 3.8.3 Comparison of energy consumption, economic benefits, and carbon emission reduction performance of insulated curing barns

As shown in **Table 6**, the heat tobacco load is similar, the heat consumption per kilogram of dry tobacco in T1 and T2 is decreases by 32.54% and 25.36%, respectively (Equation 7). Overall, the air-source heat pump-heated barns consumed one-third of the biomass-fueled barns, indicating that the coefficient of performance (COP) of the currently used air-source heat pump exceeds 3 during the tobacco curing process. T2 shortens the curing time by 14 h. The circulating fan’s electricity consumption varies by only 8.5% across biomass-fueled barns, far less than the 20.71% reduction in total biomass pellet consumption in the insulated treatment. Therefore, the circulating fan is not the main source of the overall energy savings.

**Table 6:** Comparative analysis of energy consumption among different treatments.

Treatments	Fresh tobacco weight (kg)	Dry tobacco weight (kg)	Electricity consumption (kW·h)	Biomass consumption (kg)	Curing time (h)	Heat consumption per kilogram of dry tobacco (MJ/kg)
CK1	4112.6a	714.06a	2763a	—	262b	13.93
T1	4187.2a	732.58a	2139b	—	257a	10.51
CK2	4165.5a	723.07a	682a	2133a	255a	52.94
T2	4120.6a	721.10a	624b	1687b	241b	42.23

Letters in the same column indicate significant differences at *P* < 0.05. The average coal consumption for thermal power supply in China corresponds to an energy conversion factor of 1 kW·h = 3.6 MJ. The average low calorific value of biomass pellets used in this study is 16.8 MJ/kg.

**Table 7** shows that the payback periods for the retrofit investment in T1 and T2 are approximately 2.6 and 1.6 years, respectively. These figures are based on the measured energy conservation data in this study: after the insulation retrofit of a single air-source heat pump-heated curing barn, Equation 8 indicates that approximately 362.7 kg CO_2_-eq emissions can be directly reduced per flue-curing cycle; after the renovation of a single biomass-fueled barn, approximately 173.70 kg CO_2_-eq emissions can be directly reduced per flue-curing cycle.

**Table 7:** Economic benefit and carbon emission reduction performance analysis of the IPM insulation retrofit for different curing barn treatments.

Treatments	Insulation retrofit cost per BCB (RMB)	Payback period (year)	Reduce carbon emissions per batch for each BCB (kg CO_2_-eq)	Annual curing batches per BCB (batches)	Reduce carbon emissions per BCB each year (t CO_2_-eq)
T1	4602.34	2.6	362.70	5	1.81
T2	4602.34	1.6	173.70	5	0.87

Based on the energy emission factor standards commonly used in the Chinese tobacco industry, the electricity emission factor is calculated using the average emission factor of 0.5810 kg CO_2_-eq/kW·h for China’s regional power grid, and the net emission factor for biomass pellets is set at 0.38 kg CO_2_-eq/kg. The electricity rate is 0.56 RMB/kW·h, and biomass pellets are priced at 1200 RMB/t.

## 4 Discussion

### 4.1 Effects of insulation treatment on pigments and ABA

Color is a key indicator of tobacco leaf quality. During curing, the degradation and transformation of internal pigments strongly influence leaf coloration (Wang et al., 2017b). The essence of tobacco curing is leaf dehydration and yellowing, during which large pigment molecules break down into smaller compounds that serve as aroma precursors (Meng et al., 2022). The carotenoid family includes important precursors to plant hormones, such as ABA and strigolactones (SLs). ABA, in particular, plays a crucial role in physiological and developmental processes, plant-environment interactions, and plant responses to abiotic and biotic stresses (Chen et al., 2020; Moreno et al., 2021).

This study found that after insulation improvements, chlorophyll and carotenoid degradation rates were higher in both the air-source heat pump-heated (T1) and biomass-fueled (T2) barns than in the conventional counterparts (CK1 and CK2). Moreover, at the same curing stage, the carotenoid-to-chlorophyll ratio was higher in the insulated barns, indicating that the optimized curing environment promoted pigment degradation. This ratio reflects the ease of tobacco yellowing: chlorophyll degrades more rapidly during curing, and over time carotenoids progressively dominate the total pigment pool, causing the carotenoid/chlorophyll ratio to increase. Pigment degradation depends on curing temperature, and precise temperature control facilitates this process. Smaller spatial temperature differences within the barn promote uniform pigment degradation across leaf positions (Chu et al., 2010; Song et al., 2010).

Following insulation improvements, ABA accumulated more rapidly in both T1 and T2 than in conventional barns. This pattern likely reflects the role of aldehydes derived from carotenoid oxidative cleavage as key precursors for ABA synthesis (Taylor et al., 2000) and the large quantities of ABA produced under high-temperature stress (Tao et al., 2022). During curing, high-temperature stress can activate antioxidant defense systems—including SOD, POD, and catalase (CAT)—thereby mitigating lipid peroxidation (Li et al., 2014). Furthermore, high-temperature stress may disrupt membrane protein structure and function, whereas ABA can regulate membrane protein phosphorylation to maintain normal function and prevent further damage to cell membranes (Tao et al., 2022). Preserving the structural and functional integrity of cell membranes helps reduce leaf scald and improve overall curing quality.

### 4.2 Effects of insulation treatment on physiological indicators

During curing, high temperatures generate large amounts of reactive oxygen species in plants, including superoxide anions (O_2_^-^) and hydrogen peroxide (H_2_O_2_). These species attack unsaturated fatty acids in cell membranes, triggering lipid peroxidation and the production of harmful substances such as MDA, ultimately damaging membrane structure and function (Hasanuzzaman et al., 2013). MDA content reflects the degree of oxidation in plants and serves as an indicator of stress-induced damage, making it a useful reference for assessing plant stress resistance (Liu, 2000; Sohag et al., 2020). PPO is a key enzyme in enzymatic browning during curing. During the leaf-drying stage, tobacco leaves tend to change from yellow to brown, a change attributed to lipid peroxidation, membrane damage, and direct interactions between PPO and polyphenols that lead to the formation of brown compounds (Zhao et al., 2022). POD catalyzes redox reactions between hydrogen peroxide (H_2_O_2_) or organic peroxides and various substrates, slowing membrane degradation (Nath et al., 2016). LOX, also known as lipoxidase or carotene oxidase, is widely present in higher plants. Studies have shown that higher temperatures accelerate respiration and transpiration rates, thereby activating enzymes such as NADPH oxidoreductase and LOX, which in turn contribute to membrane degradation (Qiao et al., 2022). Previous research indicates that LOX is not only crucial for carotenoid degradation in tobacco but also involved in chlorophyll degradation during curing (Leenhardt et al., 2006; Mares et al., 2021; Zheng et al., 2021). Under the action of LOX, tobacco leaf carotenoids are oxidized to produce aroma compounds such as β-damascone, megastigmatrienone, and β-ionone (Ma et al., 2022). It is necessary to clarify that the accelerated carotenoid degradation observed in insulated curing barns in this study follows a regulated enzymatic metabolic pathway rather than non-selective thermal oxidative destruction triggered by excessive, uncontrolled high temperatures. The uniform, stable temperature field inside the insulated barn eliminates local overheating spots above 55 °C that often occur at the controlled DBT point in conventional non-insulated barns, where the temperature distribution is uneven, during the late leaf drying phase. This prevents non-specific thermal decomposition of norisoprenoid aroma precursors generated by carotenoid cleavage.

The experimental results suggest that MDA content accumulates more rapidly in tobacco leaves cured in conventional barns, whereas insulation treatment significantly reduces MDA accumulation in insulated barns. The significantly reduced MDA accumulation and enhanced POD peroxide-scavenging capacity in tobacco leaves from insulated barns further indicate that intracellular reactive oxygen species levels remain within a relatively moderate physiological range throughout the curing process. This low-oxidative-stress environment prevents excessive free-radical attack, which would otherwise nonselectively degrade newly generated aroma-precursor molecules into non-volatile, non-aromatic residues. Lower MDA accumulation indicates less oxidative damage to cell membranes, facilitating the complete degradation and transformation of macromolecules (Liu et al., 2013). This apparent paradox—high LOX activity alongside reduced lipid peroxidation damage—can be explained by three coordinated mechanisms. Regarding spatio-temporal compartmentalization and substrate specificity: in insulated barns with more uniform, stable temperatures, increased LOX activity mainly targets free, plastid-stored carotenoids and early-released extramembrane polyunsaturated fatty acids, rather than membrane-bound phospholipids. The stable, mild environment prevents high-temperature spikes that could disrupt membrane compartments in uninsulated barns, which would otherwise allow LOX to peroxidize membrane lipids and produce MDA. In terms of directing substrate flow toward aroma precursors rather than membrane damage, the heightened LOX activity in insulated barns favors the oxidation of carotenoids, thereby generating these target carotenoid-derived aroma precursors. This shifts lipid oxidation away from the formation of harmful MDA toward beneficial flavor compounds. With the synergistic timing of increased POD activity for peroxide removal, our analysis shows that elevated POD levels during the critical 40–45 °C curing stage—when membranes are prone to rupture—coincide with increased LOX activity. POD quickly scavenges hydroperoxides produced as intermediates of the LOX reaction, preventing the uncontrolled lipid peroxidation chain reaction that generates large quantities of MDA and causes membrane damage. In contrast, in uninsulated barns, insufficient POD activity leads to peroxide buildup and elevated MDA levels, even at the same DBT settings. After insulation improvement, PPO activity decreased, helping maintain membrane integrity and preventing PPO from interacting with polyphenols, thereby avoiding enzymatic browning (Ma et al., 2022). In contrast, conventional barns exhibit higher PPO activity at curing temperatures of 42-54 °C, which may be one of the primary reasons for browning and leaf contamination. In insulated curing barns, however, the activities of POD and LOX were enhanced, improving peroxide clearance and preventing early membrane rupture. The upregulated LOX activity here serves as a specific biocatalyst that selectively cleaves the long-chain conjugated double-bond structure of carotenoids, generating the target aroma precursors β-damascone, megastigmatrienone, and β-ionone, rather than triggering non-selective peroxidative degradation of these small-molecule aroma compounds. The increased LOX activity promoted pigment degradation, facilitated leaf yellowing, and contributed to the formation of aroma compounds (Zheng et al., 2021; Pham et al., 2025). Taken together, this coordinated set of effects—substrate compartmentalization, directed flux toward aroma formation, and POD–LOX temporal synergy—rather than a vague description of “cellular systemic resistance” explains why insulated barns show significantly higher LOX activity and 15.3–19.7% lower MDA accumulation than their corresponding non-insulated control groups.

It is important to note that the current dataset does not include quantitative measurements of these specific carotenoid-derived aroma compounds. Instead, the improved aroma performance is inferred indirectly from the significantly higher total polyphenol content and the improved appearance of cured tobacco leaves under the insulation treatments. Future trials will include targeted GC-MS analysis of these key aroma compounds to provide direct evidence for this mechanistic pathway.

### 4.3 Effects of insulation treatment on tobacco leaf quality and curing barn performance

Compared with conventional curing barns, air-source heat pump and biomass-BCBs exhibit a more uniform temperature distribution during curing, with smaller temperature gradients within the barn after thermal insulation improvements. Moreover, temperature fluctuations inside the barns remain stable over time. During the leaf- and stem-drying stage, the temperature in the insulated barns is higher than in the control barns. Elevated curing temperature enhances tobacco leaf quality and increases aromatic compound concentration (Jia et al., 2023). Polyphenols not only influence the color of tobacco leaves but also play a key role in the composition of aroma compounds (Ji et al., 2022). After thermal insulation treatment, the total polyphenol content in the BCBs increases significantly. Under the same conditions, the polyphenol content in the tobacco leaves from the biomass-fueled barn is notably higher than that in the air-source heat pump-heated barn. This is likely due to the longer curing time in the air-source heat pump-heated barns, which promotes the oxidation of polyphenols into colored compounds (Chen et al., 2021). This may also explain why the tobacco leaves from air-source heat pump-heated barns have a deeper color compared to those from biomass-fueled barns. Following the improvement in thermal insulation, the external quality and physical properties of the tobacco leaves were significantly enhanced. At the same time, energy consumption in the curing barns decreased, benefiting tobacco growers by increasing profits and reducing costs. The reduction in energy consumption corresponds to a decrease in carbon emissions, advancing the tobacco industry toward greener, more sustainable development.

## 5 Conclusion

In summary, thermal insulation treatment of traditional BCBs with brick and concrete walls enhances curing performance by improving temperature uniformity, reducing temperature fluctuations during curing, and maintaining a stable environment that supports consistent pigment degradation in tobacco leaves. The insulated barns accelerate the degradation of chlorophyll and carotenoids and, by increasing POD and LOX activities, inhibit MDA accumulation and PPO-mediated enzymatic browning, thereby preserving membrane integrity. After thermal insulation treatment, the total polyphenol content in the tobacco leaves is significantly higher than in the control groups, with notable improvements in appearance quality and physical characteristics. The leaves display a richer color and higher oil content. The air-source heat pump-heated barns with thermal insulation reduce electricity consumption by 32.54% and lower carbon emissions, thereby providing both economic and ecological benefits. Despite these promising results, several limitations of the present study should be acknowledged. China has a vast territory, with flue-cured tobacco grown across widely distributed regions under varied ecological conditions. Follow-up research spanning multiple test sites over consecutive curing seasons, combined with durability tracking and a full life-cycle economic benefit evaluation, will further enhance the practical guidance value of this insulation renovation technology. These findings provide a theoretical foundation and a practical reference for promoting low-carbon curing technologies in cooler tobacco-growing regions and for improving tobacco leaf quality.

## Data Availability

All raw datasets generated during this study, including 18-point continuous dry-bulb temperature monitoring data across all curing stages, original measurements of pigment content, enzyme activity, and physiological indicators, the full set of cured tobacco leaf quality evaluation data, and real-time statistical records of electricity and biomass energy consumption, have been publicly deposited in the Dryad Digital Repository. The dataset can be freely accessed and downloaded via the permanent link: https://doi.org/10.5061/dryad.6q573n6fr.

## References

[B1] Bai Z. Guo D. Li S. Hu Y. (2017). Analysis of temperature and humidity field in a new bulk tobacco curing barn based on CFD. Sensors 17, 279–296. doi: 10.3390/s17020279 28146128 PMC5336120

[B2] Chen J. Li Y. He X. Jiao F. Xu M. Hu B. . (2021). Influences of different curing methods on chemical compositions in different types of tobaccos. Ind. Crops Prod. 167, 113534. doi: 10.1016/j.indcrop.2021.11353442574925

[B3] Chen K. Li G. Bressan R. Song C. Zhu J. Zhao Y. (2020). Abscisic acid dynamics, signaling, and functions in plants. J. Integr. Plant Biol. 62, 25–54. doi: 10.1111/jipb.12899 31850654

[B4] Chu D. Xie Y. Yong G. Yan X. Tong H. Liu S. (2010). Heat-induced changes of plastid pigments in tobacco laminas from different stalk positions during the flue-curing period. Asian J. Chem. 22, 2625–2634. Available online at: https://asianpubs.org/index.php/ajchem/article/view/11454.

[B5] Hasanuzzaman M. Nahar K. Alam M. M. Roychowdhury R. Fujita M. (2013). Physiological, biochemical, and molecular mechanisms of heat stress tolerance in plants. Int. J. Mol. Sci. 14, 9643–9684. doi: 10.3390/ijms14059643 23644891 PMC3676804

[B6] He Y. Chen Y. Zhong L. Lai Y. Kang Y. Luo M. . (2025). Spatiotemporal evolution of ecological environment quality and its drivers in the Helan Mountain, China. J. Arid. Land 17, 224–244. doi: 10.1007/s40333-025-0073-z30311153

[B7] He X. Liu T. Ren K. Chen J. Zhao G. Hu B. . (2020). Salicylic acid effects on flue-cured tobacco quality and curing characteristics during harvesting and curing in cold-stressed fields. Front. Plant Sci. 11, 580597. doi: 10.3389/fpls.2020.580597 33193524 PMC7661750

[B8] Ji S. Chen S. Su M. Zhang Y. Fan J. Chen Y. . (2022). Chemical compositions in flue-cured tobacco of different leaf structures. Tobacco Sci. Technol. 55, 57–64. doi: 10.16135/j.issn1002-0861.2022.0256

[B9] Jia J. Zhang M. Zhao J. Wang J. He F. Wang L. (2023). The effects of increasing the dry-bulb temperature during the stem-drying stage on the quality of upper leaves of flue-cured tobacco. Processes 11, 726. doi: 10.3390/pr1103072630654563

[B10] Jiang Z. Zhang Z. Zhang C. Zheng H. Wang W. Li S. J. . (2024). Study on maturity of one-time harvesting of four lower flue cured tobacco leaves in tobacco-rice rotation field. Crops 4, 129–138. doi: 10.16035/j.issn.1001-7283.2024.02.016

[B11] Leenhardt F. Lyan B. Rock E. Boussard A. Potus J. Chanliaud E. . (2006). Wheat lipoxygenase activity induces greater loss of carotenoids than vitamin E during breadmaking. J. Agric. Food. Chem. 54, 1710–1715. doi: 10.1021/jf052243m 16506823

[B12] Li Z. Gao Y. Lin C. Pan R. Ma W. Zheng Y. . (2018). Suppression of LOX activity enhanced seed vigour and longevity of tobacco (*Nicotiana tabacum* L.) seeds during storage. Conserv. Physiol. 6, coy047. doi: 10.1093/conphys/coy047 30279990 PMC6161406

[B13] Li H. Liu S. Yi C. Wang F. Zhou J. Xia X. . (2014). Hydrogen peroxide mediates abscisic acid-induced HSP70 accumulation and heat tolerance in grafted cucumber plants. Plant Cell Environ. 37, 2768–2780. doi: 10.1111/pce.12360 24773056

[B14] Li J. Muneer M. A. Sun A. Guo Q. Wang Y. Huang Z. . (2023). Magnesium application improves the morphology, nutrients uptake, photosynthetic traits, and quality of tobacco (Nicotiana tabacum L.) under cold stress. Front. Plant Sci. 14, 1078128. doi: 10.3389/fpls.2023.1078128 36844047 PMC9948613

[B15] Li Y. Ren K. Hu M. He X. Gu K. Hu B. . (2021). Cold stress in the harvest period: effects on tobacco leaf quality and curing characteristics. BMC Plant Biol. 21, 131. doi: 10.1186/s12870-021-02895-w 33685400 PMC7941949

[B16] Liu X. (2000). Physiological mechanisms of creeping bentgrass (*Agrostis palustris* L.) tolerance to summer stres (Manhattan, KS, USA: Kansas State University), 266.

[B17] Liu W. Yu K. He T. Li F. Zhang D. Liu J. (2013). The low temperature induced physiological responses of avena nuda L. a cold-tolerant plant species. Sci. World J. 2013, 658793. doi: 10.1155/2013/658793 23843738 PMC3693167

[B18] Ma Y. Liu P. Meng A. Deng L. Xue W. Chen F. . (2022). Comparative study of the biochemical properties of membrane-bound and soluble polyphenol oxidase from *prunus mume*. LWT Food Sci. Technol. 171, 114156. doi: 10.1016/j.lwt.2022.11415642574925

[B19] Mares D. J. Cheong J. Goonetilleke S. N. Mather D. E. (2021). Lipoxygenase in wheat: Genetic control and impact on stability of lutein and lutein esters. Foods 10, 1149. doi: 10.3390/foods10051149 34065461 PMC8160724

[B20] Meng L. Song W. Chen S. Hu F. Pang B. Cheng J. . (2022). Widely targeted metabolomics analysis reveals the mechanism of quality improvement of flue-cured tobacco. Front. Plant Sci. 13, 1074029. doi: 10.3389/fpls.2022.1074029 36523627 PMC9746875

[B21] Moreno J. C. Mi J. N. Alagoz Y. Al-Babili S. (2021). Plant apocarotenoids: from retrograde signaling to interspecific communication. Plant J. 105, 351–375. doi: 10.1111/tpj.15102 33258195 PMC7898548

[B22] Nath M. Bhatt D. Prasad R. Gill S. S. Anjum N. A. Tuteja N. (2016). Reactive oxygen species generation-scavenging and signaling during plant-arbuscular mycorrhizal and *piriformospora indica* interaction under stress condition. Front. Plant Sci. 7, 1574. doi: 10.3389/fpls.2016.01574 27818671 PMC5073151

[B23] Pham V. D. Gerlinsky M. Lettrari S. Ganzle M. G. (2025). Evolution and ecology of C30 carotenoid synthesis in *Lactobacillaceae* and application of pigmented lactobacilli in pasta production. Food Microbiol. 127, 104688. doi: 10.1016/j.fm.2024.104688 39667859

[B24] Qiao L. Gao M. Wang Y. Tian X. Lu L. Liu X. (2022). Integrated transcriptomic and metabolomic analysis of cultivar differences provides insights into the browning mechanism of fresh-cut potato tubers. Postharvest Biol. Technol. 188, 111905. doi: 10.1016/j.postharvbio.2022.11190542574925

[B25] Qin P. Wu Y. Yang C. Li Y. Chen Y. Wang Z. (2025). Numerical analysis on heat flow characteristics of tobacco curing process in intensive curing barns. Therm. Sci. Eng. Prog. 67, 104096. doi: 10.1016/j.tsep.2025.10409642574925

[B26] Sohag A. A. Tahjib-Ul-Arif M. Afrin S. Khan M. K. Hannan M. A. Skalicky M. . (2020). Insights into nitric oxide-mediated water balance, antioxidant defence and mineral homeostasis in rice (*Oryza sativa* L.) under chilling stress. Nitric. Oxide-Biology Chem. 100, 7–16. doi: 10.1016/j.niox.2020.04.001 32283262

[B27] Song Z. Li T. Zhang Y. Cao H. Gong C. Zhang W. (2010). The mechanism of carotenoid degradation in flue-cured tobacco and changes in the related enzyme activities at the leaf-drying stage during the bulk curing process. Agric. Sci. China 9, 1381–1388. doi: 10.1016/s1671-2927(09)60229-7

[B28] Song Z. Wei F. Su X. Wang Y. Fan Y. Wang J. (2021). Application of automatic control furnace for combustion of biomass briquette fuel for tobacco curing. Therm. Sci. 25, 2425–2435. doi: 10.2298/TSCI191115148S

[B29] Tao Z. Yan P. Zhang X. Wang D. Wang Y. Ma X. . (2022). Physiological mechanism of abscisic acid-induced heat-tolerance responses to cultivation techniques in wheat and maize-review. Agronomy-Basel 12, 1579. doi: 10.3390/agronomy1207157930654563

[B30] Taylor I. B. Burbidge A. Thompson A. J. (2000). Control of abscisic acid synthesis. J. Exp. Bot. 51, 1563–1574. doi: 10.1093/jexbot/51.350.1563 11006307

[B31] Timuralp C. Aras N. Sisman N. Aras H. (2025). Evaluation of optimum insulation thickness according to external wall types to be used in buildings to be reconstructed in Turkey. Appl. Sci.-Basel 15, 2497. doi: 10.3390/app1505249730654563

[B32] Wang L. Cheng B. Li Z. Liu T. Li J. (2017b). Intelligent tobacco flue-curing method based on leaf texture feature analysis. Optik 150, 117–130. doi: 10.1016/j.ijleo.2017.09.08842574925

[B33] Wang H. He Z. Guo C. (2025a). Multi-objective optimization of buildings' exterior wall insulation based on comprehensive analysis models and assessment indexes. Buildings 15, 781. doi: 10.3390/buildings1505078130654563

[B34] Wang J. Ruan X. Li P. Song Y. Liu X. W. Jia L. . (2025b). Integrated crew for heating and dehumidification of air-source heat pump for tobacco curing. Sci. Rep. 15, 38062. doi: 10.1038/s41598-025-21816-7 41168253 PMC12575827

[B35] Wang J. Song Z. Wei Y. Yang G. (2017a). Combination of waste-heat-recovery heat pump and auxiliary solar-energy heat supply priority for tobacco curing. Appl. Ecol. Environ. Res. 15, 1871–1882. doi: 10.15666/aeer/1504_18711882

[B36] Wang J. Wei F. Duan W. Zhang Y. Fan Y. Song Z. (2020). Performance of solar hot-water installations from roof-constructed solar collectors integrated with a central heating supply for tobacco curing. Thermal Sci. 24, 1845–1852. doi: 10.2298/TSCI190406072W

[B37] Wang J. Yin G. Chen X. Shen H. Duan W. (2024). Design and test of controlling frequency circulating fan system in bulk curing barn. Heliyon 10, e34688. doi: 10.1016/j.heliyon.2024.e34688 39148989 PMC11325055

[B38] Wang J. Zhang Q. Wei Y. Yang G. Wei F. (2019). Integrated furnace for combustion/gasification of biomass fuel for tobacco curing. Waste Biomass Valorization 10, 2037–2044. doi: 10.1007/s12649-018-0205-130311153

[B39] Wu Y. Li Y. Qin P. Yang C. Chen Y. Wang Z. . (2026a). Enhancing full-cycle thermo-flow uniformity in intensive curing barns via perforated horizontal plates with stepped spacing. Int. Commun. Heat Mass Transfer 172, 110622. doi: 10.1016/j.icheatmasstransfer.2026.11062242574925

[B40] Wu Y. Yan Y. Qin P. Li Y. Zhang C. Yang C. . (2026b). Synergistic optimization of full-cycle spatial heat flow distribution in intensive curing barns with vertical baffle and stepped spacing horizontal perforated plate. Int. Commun. Heat Mass Transfer 178, 111602. doi: 10.1016/j.icheatmasstransfer.2026.11160242574925

[B41] Wu J. Yang S. (2021). Modeling of the bulk tobacco flue-curing process using a deep learning-based method. IEEE Access 9, 71–79. doi: 10.1109/ACCESS.2021.311954425079929

[B42] Zhai S. Zhao Y. Chang J. Wang J. He F. Liu L. (2025). Effects of heat preservation treatment on nitrogen metabolism during tobacco curing in bulk curing barn. J. Henan Agric. Sci. 54, 175–184. doi: 10.15933/j.cnki.1004-3268.2025.06.019

[B43] Zhang Y. Li B. He Z. Wu W. Zhong J. Zhang X. (2022). Temperature field simulation and energy analysis of a heat pump tobacco bulk curing barn. Energies 15, 8655. doi: 10.3390/en1522865530654563

[B44] Zhao Y. Ma Y. Tang J. Wu S. Zhang J. Wei K. (2026). Analysis and improvement of temperature and humidity distribution in bulk curing barn for enhanced tobacco quality. Appl. Eng. Agric. 42, 237–249. doi: 10.13031/aea.16355

[B45] Zhao S. Wu Z. Lai M. Zhao M. Lin B. (2022). Determination of optimum humidity for air-curing of cigar tobacco leaves during the browning period. Ind. Crops Prod. 183, 114939. doi: 10.1016/j.indcrop.2022.11493942574925

[B46] Zheng X. Yang Y. Al-Babili S. (2021). Exploring the diversity and regulation of apocarotenoid metabolic pathways in plants. Front. Plant Sci. 12, 787049. doi: 10.3389/fpls.2021.787049 34956282 PMC8702529

